# Callous-unemotional traits and anxiety in adolescents: a latent profile analysis to identify different types of antisocial behavior in a high-risk community sample

**DOI:** 10.1186/s13034-022-00493-8

**Published:** 2022-07-19

**Authors:** Philip J. S. Michielsen, Maaike M. J. Habra, Joyce J. Endendijk, Diandra C. Bouter, Nina H. Grootendorst-van Mil, Witte J. G. Hoogendijk, Sabine J. Roza

**Affiliations:** 1 Department of Psychiatry, Erasmus University Medical Center, Dr. Molewaterplein 40, Rotterdam, 3015 GE The Netherlands; 2 Mental Health Institute, GGZ Westelijk Noord-Brabant, Halsteren, The Netherlands; 3 Forensic Outpatient Center Breda, Fivoor, Breda, The Netherlands; 4 Child and Adolescent Studies, Utrecht University, Utrecht, The Netherlands; 5 Department of Psychiatry, Epidemiological and Social Psychiatric Research Institute (ESPRi), Erasmus University Medical Center, Rotterdam, The Netherlands; 6 Netherlands Institute for Forensic Psychiatry and Psychology, The Hague, The Netherlands

**Keywords:** Adolescent, Aggression, Violence, Callous-Unemotional traits, Anxiety, Community

## Abstract

**Objective:**

Callous-unemotional (CU) traits are associated with a more severe and chronic trajectory of antisocial behavior. The present study aimed to identify different classes of CU and anxiety and to compare these classes on overt and covert antisocial behavior and several clinical correlates.

**Method:**

In a prospective high-risk cohort of adolescents (N = 679; mean age = 14.77, SD = 0.81), latent profile analysis was conducted using CU traits and anxiety symptoms as indicators, and multi-informant aggressive and rule breaking behavior as distal outcomes. Post-hoc analyses with binary logistic regression and a series of ANCOVA were performed on identified classes assessing violent aggression, property offending, and clinical correlates.

**Results:**

Three classes were found, a reference group (low CU, low anxiety; N = 500), a high CU-low anxiety group (N = 98), and an intermediate CU-high anxious group (N = 81). The high CU-low anxiety group scored highest on property offenses, while the intermediate CU-high anxious group scored highest on aggressive behavior. The intermediate CU-high anxious group scored highest on psychotic experiences, while the high CU group scored highest on internet gaming addiction problems and bullying victimization.

**Conclusion:**

These findings provide further evidence for diverse variants of CU traits in a high-risk community sample. Future prospective studies should point out whether and to what extent adolescents with CU traits with and without anxiety develop criminal careers and psychiatric disorders in adulthood.

**Supplementary Information:**

The online version contains supplementary material available at 10.1186/s13034-022-00493-8.

## Introduction

Callous-unemotional (CU) traits (e.g., a lack of remorse, decreased empathy and callousness) are associated with a more severe and chronic trajectory of aggressive antisocial behavior [1, 2]. These traits may serve as a precursor to psychopathy [3] and persistent antisocial behavioral problems in adulthood [4, 5]. Additionally, treatment effects may be lower in adolescents with antisocial behavior and CU traits compared to youth with antisocial behavior, but without CU traits, although this may also be the result of poorer premorbid functioning [6]. The importance of CU traits is further highlighted by the addition of a specifier to the diagnosis of conduct disorder in the DSM-5 [7].

The etiopathogenesis of CU traits, however, is still largely unknown. There is a growing body of literature stating that adolescents with CU traits represent a heterogeneous sample, with multiple developmental pathways. First, there is the primary variant, high in CU traits and low in anxiety, which is proposed to be a temperamental or genetically based deficit in emotion processing. Second, there is the secondary variant, high in CU traits and high in anxiety, which etiologically could be the result of childhood abuse or trauma exposure [3]. Low anxiety levels in primary CU variants appear to arise from a fearless temperament and lack of emotional response to caregivers when committing transgressions [8, 9]. Adolescents high in CU traits and low in anxiety also show reduced fear potentiated startle [10], decreased amygdala activity, as well as skin conductance in response to negative faces [11]. On the other hand, adolescents high in CU traits and high in anxiety show hyperarousal and oversensitivity to negative affect, which is a typical consequence of early childhood maltreatment and traumatization [12]. Emotional numbing and detachment may serve as protective coping strategies to adverse childhood experiences [12], reducing stress levels in the short run, but stimulating aggressive and antisocial behavior in the long run, by suppressing empathy and moral socialization [13].

The proportion of primary and secondary variants among adolescents varies widely, with higher rates of primary variants in justice-involved samples compared to clinical/high-risk community samples [3]. Yet, some studies failed to distinguish primary and secondary variants [14, 15]. Differences between studies might be attributed to different statistical procedures used to identify primary and secondary variants, most commonly person-centered clustering techniques, but also mean-split [16] or moderated regression analyses [17, 18]. Studies have also been heterogenic regarding the use of CU traits or the broad dimensional psychopathy concept, and in measurement instruments for anxiety [3].

Early identification of CU-traits is crucial to promote prevention strategies and to provide early interventions to youth-at-risk and their families. Further classification of adolescents with primary vs. secondary CU variants may also affect prognosis and treatment outcome. Since the secondary CU variant was repeatedly demonstrated to be more related to a history of child abuse and trauma than the primary variant [16, 19, 20], adolescents with secondary variants might benefit from trauma therapeutic intervention, whereas adolescents with primary variants might not. With regard to preventive strategies, we need detailed information on prevalence rates of adolescents with primary vs. secondary traits in (high-risk) community samples, as well as on the burden of disease and clinical correlates in both groups. Current evidence shows that adolescents with secondary CU variants display more overt antisocial (i.e., aggressive) behavior (e.g., physical assault) compared to adolescents with the primary variant [4, 19, 20]. There is a lack of population-based studies on CU variants and covert antisocial behavior (i.e., rule breaking behavior such as lying, cheating, arson, vandalism, and theft); in detained youth increased rates were found in adolescents with the secondary CU variant compared to the primary variant [20]. The importance of distinguishing overt and covert antisocial behavior is further demonstrated by a long-term follow-up study into adulthood, showing non-violent crime convictions are a common consequence to covert antisocial behavior in mid-adolescence, signaling a covert developmental pathway [21].

Another gap in the literature on primary/secondary CU variants concerns the association with risk of first episode psychosis. We found one study in which adolescents from a community sample with secondary CU traits exhibited more symptoms of psychoticism compared to the primary CU subgroup [19]. Adolescence is a vulnerable transition period in which individuals at risk can develop psychotic symptoms, early-onset psychosis or schizophrenia. Different phenotypes have been proposed regarding the relationship between psychotic disorders and antisocial/aggressive behavior: antisocial behavior may precede and accompany psychosis onset, aggressive behavior may start with psychotic illness progression, and/or an aggressive event may occur after many years of illness [22]. The role of CU traits in either trajectory is not clear, thus investigating associations with different CU variants at a developmental stage where transition into first episode psychosis is at stake is of particular importance.

Furthermore, to our knowledge, only one study investigated substance use and the associations with CU variants in a community sample: in a single gender study Goulter et al., found that girls with secondary CU traits showed significantly higher levels of substance use than a healthy control group without CU-traits, but differences between girls with secondary and girls with primary CU-traits were not statistically significant [23].

Correlates of primary or secondary CU traits and addictive behaviors such as internet gaming disorder have not been investigated either. In DSM-5 internet gaming disorder (IGD) has been included in section III, highlighting the need for further clarification as a diagnostic construct [7]. Internet gaming disorder is defined as a continuous and repeated involvement with video games, often leading to significant daily/educational/social disruptions. Nine diagnostic criteria have been proposed including withdrawal symptoms when not playing, tolerance, continuation of playing despite problems, and playing as an escape of adverse moods. Prevalence rates in adolescents vary between studies (1–6%) [24, 25]. IGD has been associated with a higher degree of attention/hyperactivity symptoms, emotional distress, and antisocial behavior in adolescents [26], though a lack of association with psychopathology has also been found [27]. Clarification of the potential association of IGD and CU traits could mark protective or threatening comorbidity in adolescents at risk.

Other manifestations of antisocial behavior are bullying perpetration and victimization. In a recent meta-analysis, it was found that CU traits are positively associated with bullying perpetration, and also with bullying victimization [28]. None of the included studies investigated primary and secondary variants. Disentangling associations with primary and secondary CU variants may better indicate targets for tailored interventions against bullying.

In the present study, we examined callous-unemotional traits with or without anxiety features in a high-risk community sample of adolescents, who were oversampled on the basis of self-reported emotional and behavioral problems. Our first aim was to cluster different subgroups based on various types of overt and covert antisocial behavior using latent profile analysis (LPA). We hypothesized to find a class of adolescents with antisocial behavior which would represent a primary CU variant (i.e., high level of CU traits and low level of anxious symptoms), a secondary CU variant (i.e., high level of CU traits and high level of anxious symptoms), an anxious variant (low levels of CU traits and high levels anxious symptoms), and a reference group with low levels of both CU traits and anxious symptoms. We expected the highest levels of overt and covert antisocial behavior in the secondary CU variant compared to the primary variant and the non-CU groups. The secondary aim of this study was to address existing gaps in knowledge on clinical correlates, therefore we explored whether substance use, internet gaming addiction, psychotic experiences, and bullying perpetration/victimization varied among these different CU variants.

## Method

### Participants and procedure

This study was conducted within the iBerry Study, a prospective cohort study in the Netherlands, designed to investigate the development of adolescent and adult psychiatric disorders [29]. Within a total sample of 16,736 children living in the greater Rotterdam Rijnmond area, self-reported emotional and behavioral problems in the first year of high school were assessed as part of a standard healthcare approach by completing the Strengths and Difficulties Questionnaire-Youth (SDQ-Y) [30]. All adolescents scoring in the top 15% of scores stratified by sex and a random sample of adolescents with the lowest 85% scores were approached to participate in a long-term cohort study. At baseline, 1,022 adolescents and their parent(s) provided written informed consent and visited the research center for in-depth psychiatric interviews and questionnaires. Approximately nine months after these baseline assessments, 679 adolescents (66,4% of 1,022; mean age = 14.77, SD = 0.81) completed a second questionnaire. All variables and covariates used in this study were gathered at baseline, except for the Youth Psychopathic Inventory (which was administered in youngsters approximately 9 months after baseline). Adolescents received a small monetary compensation. Researchers were blind to screening status. The Erasmus MC’s Medical Center's Medical Ethics Review Committee approved the study protocol (MEC-2015–007).

### Measures

#### Multi-informant antisocial behavior

The Dutch version of the Achenbach System of Empirically Based Assessment (ASEBA) questionnaires was used to measure emotional and behavioral problems [31–34]. The self-report measurement was obtained by the Youth Self Report (YSR). Both parents were asked to complete the analogous Child Behavior Checklist (CBCL) whereas the teachers were asked to complete the Teacher’s Report Form (TRF). These ASEBA-questionnaires use a three-point Likert scale ranging from 0 (“not true”) to 2 (“very true”). For the present study we used the multi-informant scores indicating “Rule Breaking Behavior” (covert) and “Aggressive Behavior” (overt) from both self-report (YSR) and informant-report (CBCL and TRF) measures, following the recommendations in measuring antisocial behavior in youth [35, 36]. Internal consistency (Cronbach’s α) scores on rule breaking behavior scales (0.85, 0.81, and 0.95 on YSR, CBCL, and TRF respectively), and aggressive behavior scales (α = 0.94, 0.86, and 0.95 on YSR, CBCL, and TRF respectively) are good [32]. Composite scores were calculated using all available informants, for which at least 75% of items per scale was completed. Inter-informant assessments were sufficiently correlated (*r*’s ≥ 0.239, *p* < *0.0*1) to combine them into one composite index score per scale.

#### CU traits

The Callous-Unemotional dimension of the Dutch version of the Youth Psychopathy Traits Inventory—Short Child Version (YPI-SCV, [37]) was used to measure CU traits. This self-report measure consists of 18 items, of which six items relate to the affective scale. The items were measured on a four-point Likert scale ranging from 1 (“does not apply at all”) to 4 (“applies very well”). Baardewijk et al. [37] showed good reliability (Cronbach’s α’s of 0.85 and 0.83) and validity (respectively *r* = 0.95 and *r* = 0.93 compared to the longer version and external measures of conduct problems, [38]); in our sample the internal consistency of the affective scale appeared acceptable (α = 0.70).

#### Anxiety symptoms

We used the Anxious/Depressed scale of the Youth Self Report (YSR) to quantify anxiety symptoms at baseline. The ASEBA-questionnaires demonstrated adequate reliability and validity [32], with mean alpha scores for the Anxious/Depressed scale being 0.84, 0.84, and 0.86 for the YSR, CBCL, and TRF respectively.

#### Other outcome measures: self-reported offending

In order to pinpoint types of delinquent behavior the Dutch adaptation of the Self-Reported Early Delinquency (SRED) was used to measure self-reported violent offending and property offenses in the past 6 months [39, 40]. This interview consisted of 23 items; internal consistency in our sample was acceptable (α = 0.70). Six items were considered as violent offending based on the Statistics Netherlands classification: *joining* *a* *fight,* *hitting* *someone* *in* *public,* *carrying* *a* *weapon,* *hitting* *someone* *resulting* *in* *use* *of* *medical* *care,* *robbery* and *fighting* *with* *a* *weapon*. Ten items concerning property offenses were derived from the SRED, including destroying property in school and publicly, spraying graffiti, fire setting, illegal trespassing, and stealing of products worth more than 100 euros.

#### Other clinical correlates

**Non-verbal IQ-score** was assessed using two subtests of a Dutch non-verbal IQ test: Snijders-Oomen Non-verbal Intelligent Test-Revised (SON-R 6–40). Raw test scores on the subsets ‘Analogies’ and ‘Categories’ were converted into estimated IQ-scores using norms tailored to exact age and sex. Testing non-verbal intelligence is insensitive to differences in exposure to Dutch language from early childhood onwards. The standards, internal consistency (α = 0.95), concept validity and criterion validity of the SON-R are good [41].

**Physical/Sexual Abuse** During interview sessions at the research center current caregivers (not accompanied by the adolescent) reported on the occurrence of lifetime incidents of physical and/or sexual abuse in their children. For this study, we dichotomized these traumatic childhood experiences into ‘any’ vs. ‘none’.

**Bullying victimization/perpetration** Adolescents reported bullying or being bullied by peers in a questionnaire from a Dutch population-based cohort [42]. Four items concerning bullying victimization and four items concerning bullying perpetration, e.g., by insult, spitting, slapping and social exclusion, were scored on a 5-point Likert scale ranging from 0 (never) to 4 (several times a week). Both scales showed adequate internal consistency: for the 4-item bullying victimization subscale (α = 0.82) and for the bullying perpetration subscale (α = 0.72).

**Psychotic experiences** The Prodromal Questionnaire (PQ-16) was used to measure self-reported subclinical psychotic symptoms [43]. It consists of 16 items, of which 14 concern positive symptoms and 2 concern negative symptoms to which the adolescent can agree (1) or disagree (0). Higher scores are indicative of more psychotic symptoms. In this sample internal consistency was sufficient (α = 0.77).

**ADHD-symptoms** We used the DSM-oriented scale of Attention Deficit/Hyperactivity of the CBCL (6–18) [32], which consists of 7 items. In our sample internal reliability was good (α = 0.84).

**Substance use** The Dutch adaptation of the Self-Reported Early Delinquency (SRED) was used to measure substance use. Two separate items were asked on respectively alcohol and illicit drug use in the past 6 months. Results were dichotomized into no use versus any use.

**Internet gaming addiction problems** The Video Game Addiction Test (VAT) [44] is derived from the Compulsive Internet use Scale and is a measure for Internet Gaming Disorder. The self-report scale consists of 14 items like loss of behavioral control, interpersonal conflict, preoccupation, gaming as a mood stabilizer and withdrawal symptoms when not playing. Each item can be scored on a 3-point scale (1 = never; 2 = sometimes; 3 = often). In our sample the VAT demonstrated adequate internal reliability (α = 0.87).

#### Covariates

Gender, age, ethnic origin, household income, and educational level were identified as possible confounders, based on known correlations of antisocial behavior and environmental factors [45, 46]. Background characteristics were determined at enrollment. The adolescent was classified as of non-Dutch origin if one of the parents or the child itself was born abroad. The country of birth of the child, or the country of birth of the mother decided on the categorization into Dutch, other-Western and non-Western background, according to the Dutch standard classification criteria of Statistics Netherlands [47]. Contrary to this classification, the iBerry study considers Japan and Indonesia as non-western countries. Household income was categorized into a total monthly income of < €1599, €1600—€2399, €2400—€4399 and > €4400. Educational level of the adolescent was coded as special needs/pre-vocational education, higher general secondary education, pre-university education, or combined education level.

### Statistical analyses

For response analyses, we compared baseline characteristics of adolescents for which information on the YPI questionnaire was available (n = 679) to baseline characteristics of adolescents with missing data on the YPI (n = 343), by using Mann–Whitney U tests (non-normally distributed variables) and Chi-square tests (categorical variables).

Differences in baseline characteristics between boys and girls were explored. Correlations between covariates (gender, age, ethnic origin, household income, and level of education) with all variables of interest for the LPA were performed using bivariate Pearson's or Spearman’s correlation. Variables of interest for the LPA were self-reported CU traits (YPI), self-reported anxiety symptoms (YSR, subscale Anxious/Depressed), multi-informant composite scores of rule-breaking behavior (YSR, CBCL, TRF subscale Rule Breaking Behavior), and the multi-informant composite score of aggressive behavior (YSR, CBCL, TRF subscale Aggressive Behavior). Mplus 8.0 [48] was used to conduct latent profile analysis (LPA, [49]) in order to identify meaningful subgroups of antisocial adolescents with similar patterns of CU traits and anxiety symptoms and to examine the associations between these subgroups and distal outcomes and predictors. LPA is used to identify homogenous subgroups based on participants’ patterns of response to continuous indicators (i.e., CU traits, anxiety symptoms). We specifically conducted an LPA with the manual BCH method, which is the recommended option for models with both predictors and continuous distal outcomes [49, 50]. This method has several advantages over 1-step LPA or 3-step LPA, including not having to re-calculate estimations of LPA when including covariates or distal outcomes while also avoiding shifts in latent class in the final stage that the 3-step method is susceptible to [49]. In the first step of this method a LPA is estimated using only the latent class indicator variables, with the other variables defined as auxiliary, and the BCH weights are saved. In step 2, the model with covariates and distal outcomes is specified, while controlling for the saved BCH weights from step 1. To identify the optimal number of latent classes, the Bayesian information criterion (BIC), the adjusted Lo-Mendell-Rubin likelihood ratio test (LMR-LRT), sufficient class counts to perform secondary analyses with adolescent gender, and theoretical interpretation were employed. Previous studies showed that LMR-LRT and BIC are good indicators to select the optimal number of classes [51, 52]. A smaller BIC indicates a better fit of the model, while p-values below 0.05 on the LMR-LRT indicate that the k-class model is statistically better than the k-1 class model. Except for indicators of the latent classes (i.e., CU traits and anxiety symptoms), the following other variables were entered as auxiliary variables in the model: gender (covariate), multi-informant aggressive behavior, and multi-informant rule-breaking behavior (distal outcomes) [49]. Logistic regression, (i.e., predicting class membership from covariates and outcome variables) was used in step 2 to establish between-group differences in gender, aggressive behavior and rule breaking behavior. For further class characterization with other correlates of CU variants, classes were exported to IBM SPSS Statistics Version 26. This was done because modeling all outcomes at once in the LPA would yield a too complex model for the BCH method [53]. All classes were compared on distal outcomes by using a series of one-way ANCOVA with ethnic origin and household income as dummy-coded categorical covariates and age as continuous covariate. For dichotomous outcome measures, binary logistic regression was used. All statistical tests were considered significant at the p < 0.05 level, post hoc comparisons for ANOVA and ANCOVA procedures were Bonferroni corrected.

Missing data resulted from declined interviews or unreturned questionnaires. For baseline demographics missings ranged from 0% to 6.1%, for all between-groups analyses on found classes in LPA missings ranged from 0% to 4.1%. The study sample was selected on those with complete data on the YSR anxious/depressed subscale and YPI scores, as these variables were necessary indicators to determine the CU variants. For additional analyses incomplete data on item level were handled by mean-value substitution. When > 25% of items were missing on a scale, cases were excluded pairwise from the analysis.

## Results

### Descriptive statistics and correlations

Analyses of missing data revealed that adolescents who did not return the YPI-questionnaire (n = 343) (further named 'non-responders’) were on average older, had a lower educational level, were more often of non-Dutch or unknown ethnic origin and had lower household incomes. Furthermore, they more often reported rule-breaking and aggressive behavior and had lower IQ-scores (see Table 1).

**Table 1:** Baseline demographics and antisocial behavior problem scores: non-response analyses and gender differences within the study population

	Non-responders (N = 343)	Study participants (N = 679)	Test-statistic (Missing analyses)	Boys (N = 326, 48.01%)	Girls (N = 353, 51.98%)	Test-statistic (Boys vs. girls)
Age M, (SD) year	15.50 (0.93)	14.77 (0.81)	Z = − 11.64**	14.78 (0.78)	14.75 (0.85)	Z = **− **0.65
Educational level (N, %)			**χ**^**2**^ = 26.43**			χ^2^ = 5.50
Special needs/prevocational	164 (47.8)	303 (44.6)		136 (41.8)	167 (47.3)	
Higher general	62 (18.0)	157 (23.1)		73 (22.4)	84 (23.8)	
Pre-university	31 (9.0)	155 (22.8)		81 (24.8)	74 (21.0)	
Combined	29 (8.4)	59 (8.7)		34 (10.4)	25 (7.1)	
Monthly Household income (N, %)			**χ**^**2**^ = 44.49**			χ^2^ = 11.42
€ < 1600	52 (15.1)	56 (8.2)		25 (7.6)	31 (8.8)	
€1600–2400	39 (11.3)	99 (14.5)		40 (12.3)	59 (16.7)	
€2400–4399	101 (29.4)	333 (49.0)		160 (49.1)	173 (48.9)	
€ > 4400	44 (12.8)	149 (21.9)		79 (24.2)	69 (19.5)	
Ethnic Origin (N, %)			**χ**^**2**^ = 49.76**			χ^2^ = 6.73
Dutch *N,* *(%)*	169 (49.2)	540 (79.5)		257 (78.8)	283 (80.2)	
Other western	14 (4.0)	41 (6.0)		19 (5.8)	22 (6.2)	
Non-western	73 (21.3)	78 (11.4)		38 (11.5)	59 (11)	
Callous Unemotional traits M (SD)	-	15.59 (7.82)		17.71 (7.99)	13.66 (7.15)	Z = − 6.51**
Self-report Anxiety problems M (SD)	4.83 (4.42)	5.14 (4.25)	Z = **− **0.01	3.62 (3.54)	5.74 (4.59)	Z = − 6.81**
Self-report Rule Breaking behavior, score/problems M (SD)	5.14 (3.19)	4.11 (2.87)	Z = − 4.88**	4.38 (2.74)	3.87 (2.96)	Z = − 3.01*
Self-report Aggressive behavior M (SD)	5.86 (4.24)	5.15 (3.93)	Z = − 2.37*	5.12 (3.72)	5.18 (4.12)	Z = **− **0.16
Parent-report Rule Breaking behavior, score/problems M (SD)	2.73 (3.05)	2.04 (2.32)	Z = − 3.02*	2.22 (2.44)	1.87 (2.19)	Z = **− **1.85
Parent-report Aggressive behavior M (SD)	4.77 (4.84)	4.22 (4.30)	Z = **− **1.04	4.01 (3.86)	4.42 (4.67)	Z = **− **0.51
Non-verbal IQ-score ! M (SD)	93.95 (12.73)	99.12 (13.57)	Z = − 5.29**	99.61 (13.85)	98.68 (13.34)	Z = **− **0.80

Callous-unemotional (CU) traits: YPI: Youth Psychopathic Inventory, CU subscale

Self-report anxiety: YSR Youth Self-report, anxious-depressed subscale

Self-report Rule Breaking behavior: YSR Youth Self-report, rule breaking behavior subscale

Self-report Aggressive behavior: YSR Youth Self-report, aggressive behavior subscale

Parent-report rule breaking behavior: CBCL Child Behavior Checklist, rule breaking behavior subscale

Parent-report Aggressive behavior: CBCL Child Behavior Checklist, aggressive behavior subscale

^*^p < 0.05; **p < 0.001

! Corrected for Flynn effect

Educational level, household income, and ethnic origin were missing for 62, 149 and 107 adolescents, respectively

Baseline characteristics of the study sample elected for LPA (n = 679) are also presented in Table 1. Overall, boys and girls were equally distributed. On average, girls reported higher levels of anxiety symptoms, whereas boys reported higher levels of rule-breaking behavior. We did not find differences in reported aggressive behavior between boys and girls. Correlations between potential covariates and variables of interest for the LPA were found significant for gender (males coded as 0 and females as 1) and its association with CU traits (r =—0.356, p < 0.01), anxiety symptoms (r = 0.249, p < 0.01), and with rule breaking behavior (r = -0.87, p < 0.05). None of the other demographic characteristics, such as household income or ethnic origin, were associated with both the indicators and outcome variables used in LPA. Therefore, only gender was included as a covariate in LPA.

### Class characterization

Table 2 presents class solutions of one to five classes from the LPA. The best fit was a solution with three classes, as evidenced by decreases in AIC, BIC, sufficient entropy, significant LMR-LRT, and sufficient adolescents in each group to lead to converging models with gender as class indicator and multi-informant aggressive and rule-breaking behavior as distal outcomes. Adolescents in class 1 (n = 500, 74%) could be characterized as the “reference group” with lowest CU traits (mean score 2.11; SD 1.82) and lowest anxiety symptoms (mean score 3.57; SD 2.68). Class 2 contained 98 (14%) adolescents and could be characterized as a high callous-unemotional type without secondary anxiety (“high CU group”). They scored highest in terms of CU traits (mean score 9.11; SD 2.08) and relatively low on anxiety symptoms (mean score 3.17; SD 2.38), like the reference group. Adolescents in class 3 (n = 81, 12%) could be characterized as a group of adolescents with intermediate CU traits (mean score 3.01; SD 2.54) and high levels of anxiety symptoms (mean score 13.62; SD 3.06) (“intermediate CU-high anxious group”). Total scores on the YPI also differed between the 3 groups, with the reference group (mean score 13.46; SD 6.27) and the intermediate CU-high anxious group (mean score 16.02; SD 7.22) scoring significantly lower than the high CU group (mean score 26.00; SD 7.00). ANOVAs were used to examine differences in CU traits and anxiety symptoms between the clusters to validate class membership. Classes differed regarding CU-traits, F (2, 676) = 515.79, p < 0.01, partial η2 = 0.76, with the high CU group scoring higher than the intermediate CU-high anxious group (p < 0.01) and the reference group (p < 0.01). The intermediate CU-high anxious group also scored significantly higher on CU traits than the reference group (p < 0.01). Clusters differed regarding anxiety symptoms, F (2, 676) = 496.38, p < 0.01, partial η2 = 0.60, with the intermediate CU-high anxious group scoring higher than the reference group (p < 0.01) and the high CU group (p < 0.01), whereas the high CU group and the reference group did not differ in anxiety symptoms (p = 0.397). As an additional analysis, we ran a LPA with total YPI scores instead of CU traits to see whether different subgroups of antisocial behavior could also be found for the broader construct of youth psychopathy. For results see Additional file 1.

**Table 2:** Model fit indices of LCA’s for deciding the number of classes with CU traits as predictor variable (and gender as covariate)

	1	2	**3**	4	5
Log-likelihood	**− **1917.21	**− **1827.27	**− 1760.04**	**− **1729.22	**− **1708.89
AIC	3842.42	3667.55	**3540.08**	3484.45	3449.79
BIC	3860.50	3700.19	**3585.29**	3543.22	3522.12
Adjusted BIC	3847.80	3677.96	**3553.54**	3501.94	3471.32
LMR-LRT	–	< .001	** < .001**	.08	.12
Entropy	–	.89	**.85**	.88	.83
Class counts					
1	679	589	**500**	470	406
2		90	**98**	103	109
3			**81**	101	104
4				5	55
5					5

Statistics in bold indicate the final model

*AIC* Akaike Information Criterion, *BIC* Bayesian Information Criterion, *LMR-LRT* Lo-Mendell-Rubin Likelihood Ratio Test

*p < .05, **p < .01

### Associations of CU-trait classes to antisocial behavior

Logistic regression analysis in the second step of the LPA, examining differences between classes in antisocial behavior, revealed that adolescents in the high CU group indeed showed more antisocial behavior (aggressive behavior and rule breaking behavior combined) than adolescents in the reference group (OR 2.18, 95% CI 1.39–3.39). Also, adolescents in the intermediate CU-high anxious group showed more antisocial behavior than adolescents in the reference group (OR 4.21, 95% CI 2.46–7.19). Adolescents in the intermediate CU-high anxious group showed more antisocial behavior than adolescents in the high CU group (OR 1.93, 95% CI 1.01–3.69). A series of post-hoc ANCOVAs were used to examine class differences separately for aggressive behavior and rule breaking behavior after controlling for age, ethnic origin, and household income (Table 3, upper part). Adolescents in the intermediate CU-high anxious group scored highest on aggressive behavior, whereas the adolescents in the high CU-traits group scored highest on property offending. Both groups scored higher on all different antisocial behaviors as compared to the reference group but did not differ significantly from each other on rule breaking behavior and violent offending.

**Table 3:** Results of AN(C)OVA group comparisons

	Reference group (N = 500) (Class 1)	High CU group (N = 98) (Class 2)	Intermediate CU- High anxiety group (N = 81) (Class 3)	Test statistics AN(C)OVA/logistic regression	p	Effect^a^
Antisocial behavior						
Aggressive Behavior mean (SD)^b^	0.19 (0.16)	0.25 (0.15)	0.33 (0.21)	F = 22.39	** < 0.001**	AN > CU > Ref
Rule-Breaking Behavior mean (SD)^b^	0.14 (0.12)	0.19 (0.11)	0.20 (0.14)	F = 14.38	**0.000**	CU, AN > Ref
Violent Offending mean (SD)^b^	0.60 (0.07)	1.37 (0.17)	1.07 (0.19)	χ^2^ = 51.18	**0.001**	AN, CU > Ref
Property Offenses mean (SD)^b^	1.14 (2.03)	2.28 (3.00)	1.48 (2.61)	χ^2^ = 37.66	**0.02** γ	CU > AN, Ref
Determinants						
Sex, % female	54.6	21.4	72.8	χ^2^ = 52.14	**0.000**	
Non-verbal IQ-score mean (SD)^b^	99.7 (13.8)	97.5 (12.5)	99.4 (13.8)	F = 0.97	0.38	-
Bullying victimization mean (SD)^b^	0.71 (1.67)	0.64 (2.00)	2.85 (3.76)	χ^2^ = 70.15	**0.000**	AN > CU, Ref
Bullying perpetration mean (SD)^b^	0.28 (1.02)	0.60 (1.07)	0.99 (2.30)	χ^2^ = 34.32	0.06	-
Adverse childhood experiences (% any)	20.7	31.3	33.3	χ^2^ = 9.41	** < 0.01**	AN,CU > Ref
Clinical correlates						
Psychotic Experiences score: mean (SD)^b^	2.65 (2.45)	3.26 (2.68)	6.61 (3.39)	χ^2^ = 83.83	**0.000**	AN > CU > Ref
ADHD symptoms mean (SD)^b^	3.98 (3.13)	4.38 (3.58)	4.86 (3.32)	F = 3.26	0.03*	AN > Ref
Internet gaming addiction mean (SD)^b^	4.28 (5.15)	7.53 (5.62)	5.21 (6.21)	F = 14.39	**0.000**	CU > AN, Ref
Alcohol use % yes	43.6	50.0	51.8	χ^2^ = 3.23	0.19	-
Drugs Use % yes	7.0	11.2	12.3	χ^2^ = 3.83	0.14	-

^*^Significant at the p < 0.05 level

^a^*AN *intermediate CU-high anxiety group, *CU* high CU group, *Ref* Reference group. Only pairwise contrasts with statistical significance are shown

^b^Controlling for adolescents’ age, ethnicity, and parental household income

Adjusted for age, household income and ethnic origin, adolescents in the high CU-group had an OR of 1.89 (95% CI: 1.17–3.07) compared to the reference group for violent offending. Adolescents in the intermediate CU-high anxious group had an OR of 2.44 (95% CI 1.46–4.06) for violent offending compared to the reference group. For property offenses, the high CU group had significantly higher odds than the reference group (OR 2.20, 95% CI 1.38–3.50). Differences between the reference group and the intermediate CU-high anxious group, however, did not reach statistical significance (OR 1.57, 95% CI 0.93–3.30).

### Associations of other determinants to CU-trait classes

Adolescents in the intermediate CU-high anxious group scored significantly higher on bullying victimization than the reference group and the high CU group, whereas the high CU-group did not significantly differ from the reference group. We found no indication of significant differences between groups on the bullying perpetration scale. Both the high CU-traits group and the intermediate CU-high anxious group reported more often a history of adverse childhood experiences as compared to the reference group but did not differ from each other (Table 3).

### Associations of CU-trait classes and clinical correlates

Both adolescents with intermediate CU-high anxious symptoms and adolescents with high CU traits without anxiety scored higher on psychotic experiences, compared to the reference group, with the intermediate CU-high anxious group scoring highest. ADHD-symptoms only marginally differed between groups, as the intermediate CU-high anxious group scored higher than the reference group. Adolescents with high CU traits without anxiety scored the highest on internet gaming addiction as compared to the other groups. We found no statistically significant differences between groups in substance use.

## Discussion

In the present study we investigated the presence of CU traits in a Dutch high-risk community sample of male and female adolescents and identified different subgroups based on rule-breaking and aggressive behavior and gender. Using latent profile analyses, we found three classes of adolescents that significantly differed in antisocial behavior: a high CU-low anxiety group, an intermediate CU-high anxiety group, and a reference group (low CU-low anxiety).

Contrary to our hypothesis and earlier studies in high-risk and clinical samples [16, 23, 54], a three-class- but not a four-class model- was found. This could either be the result of studying rare features like CU traits in a community sample, or of the use of two indicators (i.e., CU traits, anxiety) and one covariate (i.e., gender) in the LPA procedure. Of note is that some studies in high-risk community samples finding a fourth high anxious-low CU traits variant used a different statistical method, median-split cut-off [16] or used a female-only sample [23]. Three classes were also found in a mixed clinic-referred sample using model-based cluster analysis [20]. Our prevalence rates were comparable to those earlier described in a large community sample [4], who found figures of 7.8% for a primary CU variant, 8.9% for an anxious variant, and 2.8% for a secondary variant. Other studies dealing with primary and secondary variants in high-risk or clinical populations [17, 23, 55] used selected samples of adolescents with problem behavior or developmental adversity. Our intermediate CU-high anxiety group is likely a mixture of adolescents corresponding to the secondary CU variant, an emotionally unstable subgroup of adolescents with primarily reactive aggressive behavior, and an adolescence-limited delinquency subgroup. In sum, our results significantly add to the knowledge of detecting at-risk individuals in the community, e.g., school settings, by scrutinizing CU traits, anxiety features, and problem behavior as warning signs that call for intervention.

The intermediate CU-high anxious group yielded highest scores on aggressive behavior, whereas the scores on other types of antisocial behavior scores were comparable to the primary variant group; for property offenses the primary CU group scored highest. This combined elevated risk of both overt and covert forms in both groups is suggestive of further risk for criminal offenses, as combined overt and covert antisocial behavior is associated with high stable antisocial behavior at age 18 [56]. Although the primary CU group is often considered at prime risk for future offenses [5, 57], there is increasing evidence from longitudinal studies that antisocial behavior with comorbid anxiety symptoms confers equal risk of future mental health problems and offenses [58, 59]. Furthermore, extending previous reports [59, 60], the intermediate CU-high anxious group might experience more peer rejection, as witnessed by bullying victimization, and reactive aggression, while the primary CU group might be more prone to popularity striving and proactive aggression.

Our results further expand knowledge by showing an association between CU traits and high anxiety traits in adolescents with antisocial behavior on the one hand and psychotic experiences on the other. The intermediate CU-high anxious group reached particularly high scores on psychotic experiences. Manifestation of first-episode psychosis is in about one third of patients accompanied by aggressive behavior [61]. Common distal risk factors for disruptive behavior disorders and schizophrenia such as obstetric complications, neurodevelopmental delay and learning disabilities, together with genetic vulnerabilities, may increase risk for both behaviors. Alternatively, psychotic experiences at this age may serve as markers of a wide range of internalizing and externalizing psychiatric symptoms [62]. Though speculative, adolescents in the community with a combination of anxious symptoms, antisocial behavior and psychotic experiences could be at increased risk for future offenses or forensically relevant psychopathology. A study in convicted adults shows that a subgroup with childhood antisocial behavior and increased CU traits and anxiety are at higher odds of a diagnosis of borderline personality disorder, which can be marked by transient paranoid ideation [58].

The primary CU group scored higher than other groups on IGD. Despite contradictory results in earlier studies, a recent meta-analysis suggests IGD has been associated with several externalizing disorders, including aggressive and rule breaking behavior [63]. High CU traits can operate as both an antecedent and outcome of IGD, in that adolescents with high CU traits tend to have difficulties setting up social interactions with peers, while excessive gaming leads to avoiding real world interactions in which empathy can possibly be stimulated. Interestingly, preferred game types are differentially associated with personality features. Low empathy has been particularly found in role playing games (e.g., ‘Roblox’), whereas real world competences like leadership and self-understanding may be enhanced in Massive Multiplayer Online Role Playing Games (MMORPG) (e.g., ‘World of Warcraft’) [64]. Whether excessively playing videogames by adolescents prone to CU-traits increases the risk, severity or stability of antisocial behavior warrants further investigation.

The intermediate CU/high anxious group scored significantly higher than the other groups on bullying victimization. This finding fits with numerous studies finding exposure to abuse and trauma as a fundamental aspect of secondary variant etiology and posing individuals at risk of repeated victimization [3]. Besides, as our intermediate CU-high anxious group is no classic ‘secondary’ CU variant, adolescents reporting higher anxious symptoms without high CU traits are more likely to suffer from bullying victimization, and associated loneliness and low self-esteem [65].

The findings in this study need to be viewed in light of some limitations. First, although we included several instruments by different informants to assess antisocial behavior, we cannot exclude the risk of some reporter bias. Adolescents were the only informants on CU traits, violent and property offending, psychotic experiences and videogaming. Earlier studies revealed that adolescents with primary variant CU traits are likely to underreport severe types of antisocial behavior [20]. Second, due to selection bias, we may have tapped on a subgroup of adolescents high in CU traits but without high levels of antisocial behavior [66], as responders included in the analyses were more likely to have average intelligence, SES and were less likely to belong to ethnic minorities compared to non-responders. As a result, differences in level of CU traits and severity of antisocial behavior between the high CU and the intermediate CU-high anxious group may have been obscured. Still, our results generally fit in those of previous studies comparing aggressive behavior between primary and secondary variants in community and clinical settings [19, 20]. Third, due to the cross-sectional design, our study cannot provide direct information on the developing trajectory of (innate) CU-traits, adverse childhood experiences, anxious symptoms, other clinical markers, and antisocial behaviors. Moreover, pinpointing proximal or distal precursors over time is difficult with assessments scoping on symptoms in the previous 6 months. Caution in interpreting the results is also warranted as CU traits were measured approximately 9 months after all other assessments. Although most adolescents at mid-adolescence show stable patterns of CU traits, we cannot rule out that a small subsample either had a decreasing or increasing pattern during this time window.

In conclusion, our study provides further evidence for classification of CU variants, a high CU variant and an intermediate CU-high anxious variant, in adolescents who have not been in contact with the judicial system. Future prospective studies should point out whether and to what extent adolescents with CU traits with and without anxiety develop criminal careers and adult psychiatric disorders. Intermediate CU traits combined with high anxiety (even in the presence of high levels of aggressive behavior) may be as predictive for future delinquent behavior as high CU traits and low anxious traits. Early recognition in schools of youth with combined traumatization/anxiety symptoms and behavioral problems can be done by routine screening, though establishing cut-off levels remains challenging. These adolescents may benefit from thorough assessment, including internet gaming addiction and psychotic experiences. Moreover, the association of CU traits and internet gaming disorder needs further clarification in terms of loss of control and violent aspects of game playing. As elevated CU traits are related to reduced facial reactions of sadness and disgust to violent films [67], the question is raised to what extent violent game playing in mid-adolescence fuels the risk of later violent aggressive acts, particularly in children with primary CU-trait variants. In sum, early identification of youth with CU/anxious traits may better guide proposals for intervention.

## Supplementary Information

**Additional file 1: Table S1**. Model Fit Indices of LCA’s for Deciding the Number of Classes (with gender as covariate); total YPI instead of CU traits as predictor variable.

## Data Availability

Data are available from the authors upon reasonable request.
