# Supplementary material for: Callous-unemotional traits and anxiety in adolescents: a latent profile analysis to identify different types of antisocial behavior in a high-risk community sample
Source: Child Adolesc Psychiatry Ment Health. 2022 Jul 19;16:58. doi: 10.1186/s13034-022-00493-8 (PMC9297635; doi:10.1186/s13034-022-00493-8)
Supplement: Supplementary file 1 — Additional file 1: Table S1. Model Fit Indices of LCA’s for Deciding the Number of Classes (with gender as covariate); total YPI instead of CU traits as predictor variable. [file 13034_2022_493_MOESM1_ESM.docx]

As an additional analysis, we ran a LCA with total YPI scores instead of CU traits to see whether different subgroups of antisocial behavior could also be found for the broader construct of youth psychopathy. We found best model fit for a solution with 2 classes (AIC=3667.76; BIC=3699.41; LMR-RT <0.001) with class 1 (n=589) scoring low on anxious symptoms and YPI, while in class 2 (n=90) adolescents scored high on anxious symptoms and low on the YPI (see table). As no classes with increased psychopathy scores could be distinguished, we did not further analyze associations of these 2 classes with the other clinical variables.

Table Model Fit Indices of LCA’s for Deciding the Number of Classes (with gender as covariate); total YPI instead of CU traits as predictor variable.

|  | 1 | 2 | **3** | 4 | 5 |
| --- | --- | --- | --- | --- | --- |
| Log-likelihood | -1916.24 | **-1826.88** | -1805.04 | -1780.59 | -1761.58 |
| AIC | 3840.48 | **3667.76** | 3630.08 | 3587.17 | 3555.16 |
| BIC | 3858.56 | **3699.41** | 3675.29 | 3645.94 | 3627.49 |
| Adjusted BIC | 3845.86 | **3677.18** | 3643.53 | 3604.66 | 3576.69 |
| LMR-LRT | - | **<.001** | .17 | .02 | <.001 |
| Entropy | - | **.89** | .82 | .86 | .79 |
| Class counts |  |  |  |  |  |
| 1 | 679 | **589** | 508 | 456 | 383 |
| 2 |  | **90** | 122 | 156 | 156 |
| 3 |  |  | 49 | 60 | 77 |
| 4 |  |  |  | 7 | 56 |
| 5 |  |  |  |  | 7 |

*Note.* Statistics in bold indicate the final model. AIC = Akaike Information Criterion, BIC = Bayesian Information Criterion, LMR-LRT = Lo-Mendell-Rubin Likelihood Ratio Test.
